# Risk of Testing Positive for COVID-19 among Healthcare and Healthcare-Related Workers

**DOI:** 10.3390/vaccines11071260

**Published:** 2023-07-19

**Authors:** Eder Fernando Ríos-Bracamontes, Luz Elena Iñiguez-Arias, Rodolfo José Ochoa-Jiménez, José Guzmán-Esquivel, Martha Irazema Cárdenas-Rojas, Efrén Murillo-Zamora

**Affiliations:** 1 Departamento de Medicina Interna, Hospital General de Zona No. 1, Instituto Mexicano del Seguro Social, Av. Lapislázuli 250, Col. El Haya, Villa de Álvarez 28984, Mexico; 2 Unidad de Investigación en Epidemiología Clínica, Instituto Mexicano del Seguro Social, Av. Lapislázuli 250, Col. El Haya, Villa de Álvarez 28984, Mexicomartha.cardenasr@imss.gob.mx (M.I.C.-R.)

**Keywords:** COVID-19, pandemics, delivery of healthcare, risk

## Abstract

Understanding the risk factors associated with COVID-19 infection among healthcare workers is crucial for infection prevention and control. The aim of this study was to examine the risk of testing positive for COVID-19 among a multicenter cohort of workers, taking into account their occupational roles (medical professionals, staff in operational and administrative roles, or laboratory personnel) in healthcare settings. The data analyzed in this study included 2163 individuals with suggestive COVID-19 symptoms who underwent laboratory testing. The incidence rate in the study sample was calculated to be 15.3 cases per 10,000 person-days. The results from the multiple regression model indicated that job roles were not significantly associated with the risk of testing positive. However, age and the duration of the pandemic were identified as significant risk factors, with increasing age and longer pandemic duration being associated with a higher risk of testing positive. Additionally, vaccination was found to reduce the risk of testing positive. These findings provide valuable insights into COVID-19 transmission among indoor healthcare workers, highlighting the influence of age, pandemic duration, and vaccination on infection risk. Further research is needed to develop evidence-based strategies aimed at protecting healthcare workers and preventing virus spread in healthcare settings.

## 1. Introduction

The coronavirus disease 2019 (COVID-19) pandemic posed major challenges for healthcare systems worldwide, with healthcare workers at the forefront of the battle against this infectious disease [1]. As the primary providers, healthcare and healthcare-related workers faced an elevated risk of exposure to severe acute respiratory syndrome coronavirus 2 (SARS-CoV-2) [2,3]. Understanding the specific risk factors associated with testing positive for COVID-19 among this population is crucial for effective infection prevention and control strategies [4].

Infection transmission of SARS-CoV-2 within healthcare settings is concerning due to its potential for rapid dissemination and increased risk to vulnerable individuals [5]. Several factors contribute to the airborne transmission of pathogens in healthcare facilities, and they include, among others, the incorrect use of face masks and other protective measures as well as inadequate ventilation systems and overcrowded spaces [6,7]. These latter may facilitate the accumulation and prolonged suspension of infectious aerosols, thereby enhancing the risk of transmission.

Numerous studies have focused on the risk of COVID-19 among healthcare workers, but most have primarily examined short-term trends or limited timeframes within the pandemic [8,9]. To comprehensively assess the risk over a more extended period, we conducted an analysis of COVID-19 testing outcomes among healthcare and healthcare-related workers. In accordance with normative standards in Mexico, where this study was conducted, molecular diagnosis (reverse-transcription polymerase chain reaction, RT-PCR), once rapid antigenic testing became available (which occurred by the last quarter of 2020), was reserved for hospitalized patients [10].

This study aimed to investigate the risk of testing positive for COVID-19 among a group of workers, considering their job roles and other relevant conditions. By analyzing a diverse range of healthcare-related facilities, we strive to provide a comprehensive understanding of the risk factors and dynamics of COVID-19 transmission in healthcare environments [11]. We acknowledge the limitations of serological testing in diagnosing SARS-CoV-2 infection. Therefore, considering the participants who were diagnosed using this method, we emphasize that our focus was on the risk of testing positive for COVID-19, irrespective of their actual condition (which would be evaluated using molecular diagnosis).

The findings from this analysis could contribute valuable insights to inform evidence-based strategies for protecting the health and well-being of healthcare workers and reducing the spread of COVID-19 within healthcare settings.

## 2. Materials and Methods

We conducted a retrospective, closed, and multicenter cohort study from March to May 2023 in the state of Colima, located in western Mexico and along the central Pacific coast. The study took place in medical units at both the primary level (n = 9) and secondary level (n = 2) of healthcare, all of which are part of the Mexican Institute of Social Security (IMSS), a component of Mexico’s public healthcare system. The IMSS provides comprehensive medical and social services to approximately 62% of the state’s total population [12]. These services are delivered by 4293 workers across various categories, including medical professionals (such as physicians, nurses, and dentists), laboratory personnel, and staff in operational and administrative roles (e.g., secretaries, cleaning staff, and social workers, among others).

Corrected version: All employees of the IMSS who actively worked in any enclosed area between March 2020 and February 2022 and presented suggestive symptoms of COVID-19 (fever, cough, sore throat, rhinorrhea) were considered eligible for the study if they underwent laboratory testing. Workers with outdoor activities (primarily administrative personnel, such as field supervisors) were excluded. Enrolled individuals were followed up until the primary binary outcome occurred, which was the laboratory test result for COVID-19 categorized as negative or positive. Only the first symptomatic episode of the disease (confirmed or discarded) was included in the analysis.

Potential eligible subjects were identified using a comprehensive normative and web-based system that was implemented for the epidemiological surveillance of respiratory viral pathogens within the IMSS [13]. This system collects extensive data on suspected cases of COVID-19, including demographic information, relevant medical records (including personal history of chronic non-communicable diseases), employment status (IMSS employee, yes/no, and specific job title), and COVID-19 vaccination status. In this study, vaccinated individuals were defined as those who had received at least one dose of any COVID-19 vaccine at least 15 days prior to the onset of symptoms. The primary data sources for this system are medical records, and when appropriate, death certificates are incorporated to ensure continuous monitoring of cases until the final disease outcome is determined.

The diagnosis of COVID-19 in accordance with normative standards was established through nasal swabbing (with a sensitivity and specificity of approximately 46% and 95%, respectively) [14] and molecular diagnosis using RT-PCR or rapid antigenic testing for severe and non-severe cases, respectively.

A comprehensive description of the laboratory methods for molecular and antigenic testing employed at the institute where the study was conducted has been published elsewhere [14,15].

**Figure 1:**
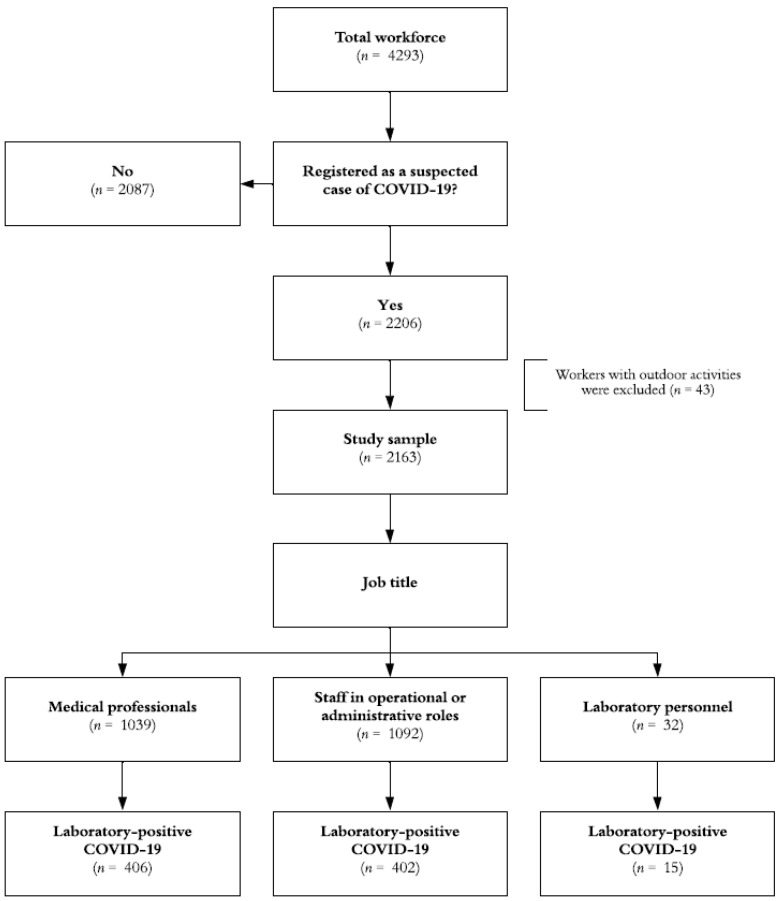
Study flowchart, Mexico 2020–2022.

The outcome of interest, along with information on job roles in the indoor healthcare environment (including medical personnel, laboratory staff, and administrative roles), as well as relevant details regarding the personal history of enrolled subjects, date of symptom onset, and vaccination status, were obtained from the audited surveillance system’s dataset. A summary of the study flowchart is provided in Figure 1.

After identifying the eligible population, we implemented a quality control procedure to verify the employment relationship of each individual with the IMSS. This mechanism involved cross-checking their affiliation with the support of the human resources department.

Summary statistics were computed, and the significance level (α) was set at 5%. We used risk ratios (RR) and 95% confidence intervals (CI), computed through generalized linear regression models, to evaluate the effect the of job role in the indoor healthcare environment on the risk for testing positive (RT-PCR or rapid antigenic testing) for COVID-19.

This study was reviewed and approved by the Local Committee of Ethics in Health Research (601) of the IMSS (approval R-2023-601-011). None of the participants were physically located or interviewed during any stage of this study, and all researchers adhered to strict ethical guidelines.

## 3. Results

Data from 2163 individuals engaged in job-related activities, presenting respiratory symptoms, and undergoing COVID-19 testing, were analyzed, with a total follow-up period of 537,552 person-days. These individuals represent approximately half of the total workforce at the IMSS facility where the study was conducted.

We observed 823 laboratory-confirmed COVID-19 cases, resulting in an overall incidence rate of 15.3 per 10,000 person-days within our study sample. However, this incidence rate varied among different job roles, with rates of 15.8, 14.7, and 28.8 observed among medical professionals, staff in operational and administrative roles, and laboratory staff, respectively. When considering the entire workforce (n = 4239) as the denominator, the overall incidence rate of the disease was 2.2 per 10,000 person-days. When measured as risk or cumulative incidence, the estimates were 39.1%, 36.8%, and 46.9% among medical professionals, personnel in operational or administrative roles, and laboratory staff, respectively. Among the enrolled participants, 6.4% (n = 53/823) were confirmed positive through RT-PCR testing, while the remaining cases were confirmed using rapid antigenic testing. Only two participants required hospital admission.

Approximately two-thirds of the participants were female (65.4%), and the mean age of the enrolled subjects was 36.4 ± 8.0 years (mean ± standard deviation). Based on job titles, the majority of participants (50.5%) held operational and administrative roles, followed by medical professionals (48.0%) and laboratory staff (1.5%). The group of medical professionals comprised 670 nurses (64.4%), 358 physicians (34.5%), and 11 dentists (1.1%). Other characteristics of the study sample, including the results of COVID-19 testing, are presented in Table 1. Participants who tested positive were older and more likely to be unvaccinated.

In the multiple generalized linear regression model (Table 2), we found no significant association between job roles and the risk of testing positive for COVID-19. Compared to medical professionals, the relative risk (RR) was 0.97 (95% CI 0.93–1.01, p = 0.130) for staff in operational and administrative roles and 1.10 (95% CI 0.94–1.30, p = 0.245) for laboratory staff. Additionally, in the multiple model, we documented that age was associated with an increased risk of testing positive for COVID-19 (per each additional year of age: RR = 1.003, 95% CI 1.001–1.006, p = 0.012), as well as more advanced pandemic development (per each additional week since the beginning of the pandemic: RR = 1.008, 95% CI 1.007–1.009, p < 0.001).

When compared with unvaccinated subjects, those who were passively immunized had a slightly but significantly reduced risk of testing positive (RR = 0.95, 95% CI 0.91–0.99, p = 0.015). No other significant associations were documented.

## 4. Discussion

The findings of this study provide valuable insights into the relationship between job roles and the risk of testing positive for COVID-19. Our analysis included a diverse cohort of 2163 individuals, with a substantial follow-up period of 537,552 person-days. Despite the variation in job roles and exposure levels, we found no significant association between job roles and the risk of COVID-19 infection.

Most participants in this study were staff in operational and administrative roles, comprising 50.5% of the cohort. These individuals play essential roles in supporting the functioning of various settings, such as healthcare facilities and administrative offices. Contrary to expectations, we did not observe a reduced risk of COVID-19 infection among personnel in operational and administrative roles when compared to medical professionals. This finding suggests that appropriate infection control measures and preventive strategies implemented in these settings have effectively mitigated the risk of transmission [16].

On the other hand, medical professionals represented 48.0% of the study population, and it is noteworthy that they exhibited a slightly lower incidence rate of COVID-19 compared to personnel in operational and administrative roles. This finding might be attributed to the heightened awareness and adherence to infection control protocols among healthcare workers, along with their access to personal protective equipment (PPE) and extensive training on infection prevention and control [17,18]. It is important to acknowledge the dedication and efforts of medical professionals in maintaining a lower risk of COVID-19 infection despite their direct exposure to infected individuals.

In contrast, laboratory staff accounted for a small percentage (1.5%) of the study population but had the highest observed incidence rate of COVID-19. These individuals are responsible for handling and processing samples, which may involve a higher risk of exposure to the virus. The elevated incidence rate observed among laboratory staff highlights the need for stringent safety measures and regular testing protocols within laboratory settings to minimize the risk of transmission and protect this vulnerable group of workers [19].

We also observed, in the bivariate analysis, that the proportion of vaccinated subjects was higher among individuals with a positive test result compared to those who were negative (56.7% vs. 50.5%, p = 0.004). We hypothesize that this finding may be associated with risk-compensatory behaviors, such as increased socialization during lockdown and misuse of non-pharmacological interventions such as facemasks, among vaccinated individuals, as has been previously described [20]. However, in the multiple model, we found that vaccination was associated with a reduced risk of testing positive (RR = 0.95, 95% CI 0.91–0.99, p = 0.015).

To the best of our knowledge, there are limited published data evaluating the specific impact of job roles in healthcare settings on the risk of testing positive for COVID-19, and the findings are heterogeneous. Two studies conducted in Germany reported an increased risk of testing positive for nurses and physicians compared to non-patient care occupations [4,21]. Additionally, a separate study indicated a higher risk among administrative personnel [22]. We acknowledge that these results may vary due to factors such as the rate of community transmission of SARS-CoV-2 in the study settings [23,24] and the indoor characteristics, including ventilation, of healthcare facilities [25].

Our study also demonstrated the impact of age on the risk of testing positive for COVID-19. With each additional year of age, the risk of infection increased slightly [26,27]. Consequently, age should be considered a significant factor when developing targeted prevention strategies and prioritizing vaccination efforts [28,29,30].

Furthermore, our analysis revealed a significant association between the duration of the pandemic and the risk of testing positive for COVID-19. As the number of infected individuals rises with the progression of the pandemic, the overall prevalence of the virus in the community increases, increasing the likelihood of individuals coming into contact with infected individuals [31]. Additionally, the reporting of more cases may indicate a higher level of community transmission, suggesting active spread of the virus within the population [32]. This finding highlights the critical importance of implementing sustained public health measures, continuous surveillance, and early detection strategies to effectively control the spread of the virus [33].

Importantly, our study investigated the impact of passive immunization on the risk of testing positive for COVID-19. Individuals who received passive immunization demonstrated a slight yet significant reduction in the risk of infection compared to unvaccinated subjects. This finding aligns with previous research conducted in this and other populations [34,35,36,37], emphasizing the potential advantages of vaccination strategies in mitigating the risk of severe disease outcomes and transmission [38].

It is crucial to acknowledge the limitations of our study. Firstly, our analysis was restricted to symptomatic infections and those recorded in the surveillance system. As a result, all asymptomatic infections and cases that were not accurately registered for surveillance or statistical purposes were omitted. Secondly, our findings are based on data from a single state in Mexico, which may limit their generalizability to other settings or populations. Thirdly, the study design was observational, and despite adjusting for several confounding factors in our regression models, the possibility of residual confounding cannot be completely eliminated. To further understand the relationship between job roles and the risk of COVID-19 infection, future studies with larger sample sizes, multicenter designs, and comprehensive assessments of job roles and exposures are warranted.

Fourthly, it is important to acknowledge the limitations associated with rapid antigenic testing, which constituted the primary (97.5%) method of participant inclusion in this study. This occurred because, during the COVID-19 pandemic, normative standards reserved molecular diagnosis for hospitalized patients, and most of the analyzed participants received ambulatory management [13]. Rapid antigen tests have demonstrated lower sensitivity compared to the gold standard RT-PCR assay, thereby increasing the likelihood of false-negative results. This reduced sensitivity is particularly evident in individuals with lower viral loads or during the early stages of infection. Additionally, the specificity of rapid antigen tests can vary, leading to potential false-positive results. On the other hand, the specificity of these tests is high, so the false-positive rate is low. Factors such as cross-reactivity with other respiratory viruses or non-specific binding can contribute to inaccurate outcomes [39]. The performance of rapid antigen tests is also influenced by the quality and handling of the test kits, as well as the expertise of the testing personnel [40]. Variability in these factors can significantly impact the accuracy and reliability of the results. Thus, it is important to interpret the findings from rapid antigenic testing with caution and consider confirmatory testing using RT-PCR to ensure accurate diagnosis, especially in cases with high clinical suspicion or when precise and sensitive detection is crucial for appropriate management and implementation of effective infection control measures.

## 5. Conclusions

Our results suggest that job roles, specifically personnel in operational and administrative roles, medical professionals, and laboratory staff, do not significantly influence the risk of testing positive for COVID-19. However, age and the duration of the pandemic were associated with an increased risk of infection. These findings emphasize the importance of implementing robust infection control measures, targeted preventive strategies, and vaccination campaigns to protect individuals across different job roles and age groups. Continued research and surveillance efforts are crucial to inform evidence-based policies and interventions for mitigating the impact of COVID-19.

## Figures and Tables

**Table 1:** Characteristics of the study sample for selected variables, Mexico 2020–2022.

Characteristic	COVID-19 ^1^ Test		p	Follow-Up (Days)
	Negative n (%)	Positive n (%)		
Gender				
Female	871 (65.0)	543 (66.0)	0.642	355,509
Male	461 (35.0)	280 (34.0)		182,043
Age (years, mean ± SD ^2^)	36.0 ± 7.9	37.0 ± 8.2	0.004	537,552
Job role				
Medical professional	633 (47.2)	406 (49.3)	0.328	256,205
Operational or administrative roles	690 (51.5)	402 (49.9)		274,128
Laboratory staff	17 (1.3)	15 (1.8)		7219
COVID-19 vaccination status				
Unvaccinated	664 (49.5)	356 (43.3)	0.004	202,180
Vaccinated	676 (50.5)	467 (56.7)		335,372
Personal history of:				
Tobacco use, yes	75 (5.6)	35 (4.3)	0.167	24,577
Obesity (BMI ^3^ ≥ 30), yes	194 (14.5)	113 (13.7)	0.629	70,882
Asthma, yes	57 (4.3)	32 (3.9)	0.678	21,905
COPD ^4^, yes	3 (0.2)	2 (0.2)	0.928	1012
Type 2 diabetes mellitus, yes	3 (3.7)	32 (3.9)	0.783	18,265
Arterial hypertension, yes	103 (7.7)	61 (7.4)	0.815	40,308
Immunosuppression (any cause), yes	5 (0.4)	1 (0.1)	0.280	1348

^1^ Coronavirus disease 2019; ^2^ standard deviation; ^3^ body mass index; ^4^ chronic pulmonary obstructive disease. Notes: (1) the absolute frequencies (n) and relative frequencies (%) are presented, unless specified as the arithmetic mean; (2) Chi-squared test *p*-values are reported, except for the age variable, where the t-test is used.

**Table 2:** Factors associated with the risk of testing positive for COVID-19 ^1^, Mexico 2020–2022.

Characteristic	$\mathrm{RR}{\;}^{2}\;(95\%\;\mathrm{CI}),\;p$	
	Bivariate Analysis	Multivariate Analysis
Gender		
Female	1.00	1.00
Male	0.99 (0.95–1.03), 0.643	0.99 (0.96–1.04), 0.994
Age (per each additional year)	1.004 (1.001–1.006), 0.004	1.003 (1.001–1.006), 0.012
Job role		
Medical professional	1.00	1.00
Operational or administrative roles	0.98 (0.94–1.02), 0.282	0.97 (0.93–1.01), 0.130
Laboratory staff	1.08 (0.91–1.28), 0.371	1.10 (0.94–1.30), 0.245
COVID-19 vaccination status		
Unvaccinated	1.00	1.00
Vaccinated	1.06 (1.02–1.16), 0.004	0.95 (0.91–0.99), 0.015
Date of symptom onset (measured in weeks since the start of the pandemic)	1.008 (1.006–1.009), <0.001	1.008 (1.007–1.009), <0.001
Personal history of:		
Tobacco use		
No	1.00	1.00
Yes	0.94 (0.85–1.03), 0.167	0.98 (0.89–1.07), 0.591
Immunosuppression (any cause)		
No	1.00	1.00
Yes	0.81 (0.55–1.19), 0.280	0.84 (0.92–1.12), 0.785

^1^ Coronavirus disease 2019; ^2^ relative risk. Notes: (1) generalized linear regression models were used to obtain the reported estimates; (2) the estimates from the multiple regression model were adjusted for all the variables presented in the table.

## Data Availability

The data and materials analyzed in this study are available from the corresponding author upon request.
